# Mechanisms and Application of Gas-Based Anticancer Therapies

**DOI:** 10.3390/ph16101394

**Published:** 2023-10-02

**Authors:** Peng Ji, Kexin Yang, Qingqing Xu, Guilin Qin, Qianyu Zhu, Ying Qian, Wenshui Yao

**Affiliations:** 1 College of Pharmacy and Chemistry & Chemical Engineering, Jiangsu Provincial Key Laboratory of Chiral Pharmaceutical Chemicals Biologically Manufacturing, Taizhou University, Taizhou 225300, China; 2 Department of Anesthesiology, Fujian Maternity and Child Health Hospital, College of Clinical Medicine for Obstetrics & Gynecology and Pediatrics, Fujian Medical University, Fuzhou 350001, China

**Keywords:** gas therapy, hydrogen, carbon dioxide, nitric oxide, carbon monoxide, hydrogen sulfide, sulfur dioxide, cancer

## Abstract

Cancer is still one of the major factors threatening public health, with morbidity and mortality rates at the forefront of the world. Clinical drawbacks, such as high toxicity and side effects of drug therapy, and easy recurrence after surgery affect its therapeutic effect. Gas signaling molecules are essential in maintaining biological homeostasis and physiological functions as specific chemical substances for biological information transfer. In recent years, the physiological regulatory functions of gas molecules in the cancer process have been gradually revealed and have shown broad application prospects in tumor therapy. In this paper, standard gas therapies are classified and introduced. Taking H_2_, CO_2_, NO, CO, H_2_S, and SO_2_ gases as examples, the research progress and application of gas therapies in malignant tumors are mainly introduced in terms of biological characteristics, anticancer mechanisms, and treatment strategies. Finally, the problems and prospects for developing gases as anticancer drugs are outlined.

## 1. Introduction

As a disease with a high mortality rate and low cure rate, cancer (malignant tumors) poses a severe threat to human health, and there is an urgent need to develop safe and effective treatment strategies [1]. The etiology of cancer—such as external habits and environmental factors as well as congenital heredity or acquired mutations and other factors—has not been very clear [2]. The clinical manifestations of cancer differ according to the tissues and organs affected, as well as the rate of deterioration. However, the biological features of cancer are fundamentally similar and include proliferation, abnormal differentiation, infiltrative growth, and strong metastatic capability [3]. Currently, clinical treatments for malignant tumors comprise surgical resection, radiotherapy, and chemotherapy. Surgical resection is the preferred treatment for patients in the early stages of cancer, while palliative surgery is required if the tumor develops infiltration and metastasis. This surgery can remove the primary and metastatic foci, improving the quality of survival for the patient to a certain extent. Radiotherapy and chemotherapy can effectively cure tumors, albeit with a high risk of severe adverse reactions [4]. In particular, tumor patients have suffered from resistance to chemotherapeutic drugs to varying degrees during antitumor drug therapy, and multidrug resistance (MDR) is one of the main reasons for the failure of antitumor chemotherapy [5]. Multidrug resistance refers to the resistance of tumor cells to one antitumor drug while simultaneously generating cross-resistance to antitumor drugs with different structures and mechanisms of action, which is a significant problem plaguing the clinical practice of medical oncology. Therefore, there is an urgent need to find a flexible and less harmful therapeutic strategy to break through the bottleneck of malignant tumor treatment [6].

**Figure 1:**
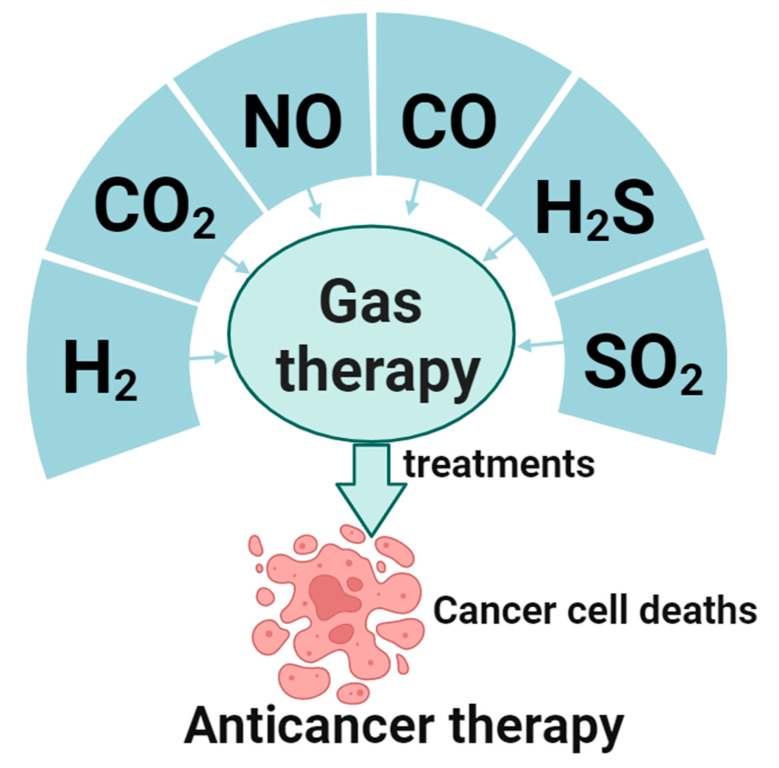
Gases for gaseous anticancer therapy and their applications.

Gas therapy is receiving increasing attention as a therapeutic approach in cancer treatment, owing to its benefits of nonresistance, low toxicity, short treatment duration, and synergy with chemotherapy and radiotherapy [7]. The living system contains multiple gas molecules that play vital roles in the body. Of these, H_2_, CO_2_, NO, CO, H_2_S, and SO_2_ have been widely acknowledged as gaseous signaling molecules that transmit signaling pathways [8,9]. Such molecules point toward the potential of gaseous signaling in cancer therapy. These gases exist in the human body as trace elements, serving as signaling molecules in various pathways. Moreover, their by-products have minimal to no side effects. Gas therapy is a burgeoning field, and the safe, effective implementation of these gases in cancer treatment can become a “green therapy” for application in biomedical and clinical treatment and other vital areas [8]. This article presents the most recent global research findings and an overview of gas therapy’s current progress (Figure 1), which offers innovative possibilities for the future of gas-based anticancer treatments.

## 2. Hydrogen

Hydrogen is the lightest element in the periodic table, predominantly found in its gaseous form, colorless, transparent, and low-density. Most of its monomers exist as hydrogen gas [10]. It is well known that the element hydrogen, located first on the periodic table, is the lightest element known, and most of its monomers are in the form of hydrogen gas, which is colorless and transparent and has a low density. In a study of the potential of hydrogen as an antioxidant, Ohsawa et al. found that hydrogen can selectively reduce hydroxyl radicals, reduce damage from oxidative stress, and have a protective effect on cells [11]. On this basis, it has been gradually discovered that hydrogen has a therapeutic impact on oxidative damage, inflammatory responses, and apoptosis caused by various diseases. The selective antioxidant effect of hydrogen can also be applied to the treatment of cancers and has led to some breakthroughs and improved some of the drawbacks of traditional malignant tumor treatment. The paper will summarize some of the substantial advances in treating cancers with hydrogen, analyze the advantages and possible disadvantages, and look forward to the future use of hydrogen as a therapeutic agent for cancers.

### 2.1. Possible Mechanisms and Signaling Pathways of Hydrogen’s Antitumor Effects

Angiogenesis in tumor development and oxidative stress are inextricably linked. ROS damage intracellular DNA, proteins, and lipids in cell membranes, resulting in cellular carcinogenesis. Due to selective antioxidants, hydrogen has a significant inhibitory effect on tumor control [12]. Animal models of hepatocellular carcinoma (HCC) have shown that hydrogen molecules can significantly reduce the incidence of hepatocellular carcinoma, decrease tumor volume, and reduce oxidative stress [13]. Hydrogen-enriched water significantly inhibits renal carcinoma induced by ferric nitrilotriacetate in Wistar rats [14]. Hydrogen-rich water significantly inhibited inflammatory effects, vascular endothelial growth factor (VEGF) expression, and signaling transcriptional activator in rat kidney 3-phosphorylation. It significantly inhibited the expression of proliferating cell nuclear antigens [13]. Frajese found that hydrogen-enriched electrolyzed water induced apoptosis in breast cancer cells and inhibited the expression of tyrosine kinase receptor 2 (ErbB2/neu), indicating that hydrogen-enriched electrolyzed water did not significantly affect the extracellular regulation of protein kinases (ERK1/2) and protein kinase B (AKT) [15]. Pyroptosis belongs to a novel inflammatory programmed cell death pathway, and its possible prognosis is related to the execution of the terminal protein cell pyroptosis managers (GSDMD). Hydrogen plays a dual effect in cancer cells: it promotes apoptosis and protects normal cells, which may trigger GSDMD pathway-mediated thermal prolapse, which can be further developed as a sensitizer for GSDMD-targeted therapies, and thus, hydrogen may initiate GSDMD pathway-mediated pyroptosis concerning inflammation-dependent cell death mechanisms [16].

The only antioxidant theories currently available cannot accurately explain the antitumor mechanism of hydrogen for the time being, and it is understood that the involvement of hydrogen is only biological. The most direct evidence of physiological activity is its antioxidant activity; in any case, satisfactory clinical results have been achieved [11]. It is worthwhile to mention that hydrogen medicine research has shown that hydrogen is involved in regulating signaling pathways that can affect protein structure and enzyme activity, which may provide new ideas for hydrogen antitumor research. In addition to exerting a direct antioxidant effect, hydrogen modulates antioxidant enzymes. Hydrogen can fight tumors by improving the body’s immune system. As coenzyme Q10 levels rise, more hydrogen molecules enter the interior of the mitochondria to function, and the patient’s overall survival is significantly improved [12]. The above study suggests that hydrogen is not unique in its ability to act through direct antioxidant effects. The above research indicates that the direct antioxidant effect is not the only ability of hydrogen; in addition to the selective antioxidant effect, hydrogen also has many other signaling molecules that play a role, so the antitumor mechanism of hydrogen needs further in-depth research.

### 2.2. Progress in Research

#### 2.2.1. Effects of ROS on Tumors

**Figure 2:**
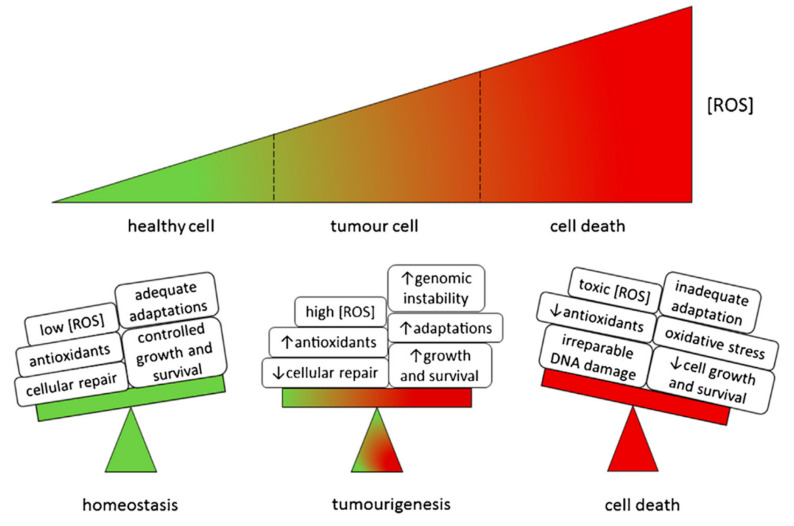
Model of ROS effects in cells. Reproduced with permission from ref. [19].

ROS comprise various aerobic radicals, consisting of superoxide anion (O^2−^), hydrogen peroxide (H_2_O_2_), hydroxyl radical (OH^−^), ozone (O_3_), and monoclinic oxygen (^1^O_2_). The unpaired electrons contained within ROS make them extremely reactive [17]. ROS are naturally produced by various biochemical processes in the organelle endoplasmic reticulum, mitochondria, and peroxidase. It is a production byproduct of the cell’s mitochondria and other cellular components [18]. Based on the current research on ROS, some antitumor strategies are as follows (Figure 2).

#### 2.2.2. Antitumor Strategies to Reduce ROS

It has been demonstrated by animal experiments that antioxidants inhibit ROS-induced oxidative DNA damage and suppress the growth of tumor cells. Two different antioxidant substances exist in the human body: one is enzymatic and the other is nonenzymatic. They both constitute the body’s internal and external antioxidative stress protection systems and inhibit oxidative stress by scavenging excess ROS in concert with each other. While enzymatic antioxidants include several of the enzymes mentioned above, antioxidants that are not enzymes mainly include vitamin C, E, pyruvate, and taurine. However, not all antioxidants act to block the ROS response [18]. Vitamin E and selenium intake, for example, can delay the onset of tumors; the effect when they are ingested in combination is not always good. According to a survey, taking beta-carotene and vitamin A may lead to lung cancer [20]. In summary, the above studies illustrate that antioxidants’ mechanism of tumor inhibition is very complex and variable, and the specific application is subject to further observation and testing.

#### 2.2.3. Antitumor Strategies to Elevate Reactive Oxygen ROS

Because tumor cells contain higher levels of ROS than normal cells, tumor cells are more sensitive to the accumulation of ROS. ROS metabolism’s reduced GSH and thioredoxin pathways are critical antioxidants [21]. Through a deeper understanding of these metabolic pathways, better therapeutic regimens can be designed to reduce their adverse effects and inhibit tumor resistance.

### 2.3. The Effect of Inflammation on Tumors

Inflammation is a symptom of tissue edema and oozing caused by bacteria, viruses, mycoplasma, and chlamydia. Simply put, it is the defense of living tissue against injurious factors called “inflammation”.

There are two main types of inflammation formation, one of which is the acute phase. The vascular system causes acute inflammation, mainly redness, swelling, and pain. Local vasodilatation, plasma, neutral leukocytes, and so on will enter the body with the vein as the center. This is the body’s natural protection against damaged cells, viruses, and other harmful stimuli, and it rapidly activates and promotes the body’s self-repair. In this procedure, white blood cells, lymphocytes, and the chemicals they produce, such as antibodies and cytokines, are discharged into the blood or infected tissue to fight off foreign pathogens or inflammatory responses. The reduction of inflammation is not a passive inflammatory response; it is an actively programmed process that includes many cells and anti-inflammatory factors that promote the reduction of the mediators of regression [22].

In the medical field, many examples of inflammation associated with tumors, such as stomach cancer, herpes virus infection, nasopharyngeal cancer, hepatitis virus infection, and systemic inflammation (e.g., obesity, depression), have been connected to cancer incidence and poor cancer treatment outcomes. Hepatitis B is also a high-risk factor for liver cancer, with 80% of liver cancers linked to the hepatitis B virus [23]. Esophageal glandular epithelial hyperplasia, chronic pancreatitis, etc., are important factors leading to esophageal and pancreatic cancer. At the same time, the inflammatory response caused by chemotherapy has a certain tumor-treatment effect. The link between inflammation and tumors is twofold: the first is through an exogenous pathway, by which inflammation-induced (e.g., inflammatory bowel disease) response raises the risk of cancer; the second is the endogenous pathway that leads to inflammation and tumorigenesis through genetic changes (e.g., oncogenes). What can be done to counteract the role of chronic inflammation in tumors? First, nonspecific anti-inflammatory drugs inhibit chronic inflammation and tumor growth and enhance the killing of tumor cells by other therapies; second, inhibition of inflammatory signaling pathways by antibodies can effectively address specific inflammatory mediators that arise in the pathogenesis and treatment of tumors. In addition, the immunosuppressive effects of immune cells, inflammatory mediators, and cytokines on the immune system of TME can enhance the efficacy of treatments such as chemotherapy, radiotherapy, and immunotherapy [24].

#### 2.3.1. Effects of Hydrogen and ROS on Inflammation

The primary etiology of chronic inflammation is due to the persistence of inflammatory factors and tissue damage. Chronic inflammation is difficult to recognize by the immune system, and prolonged inflammation leads to aging of the organism and tissue lesions. Chronic inflammation is mainly due to untimely or incomplete treatment, and the disease progresses gradually, in some cases, for more than a few months or even several years. Chronic inflammatory cell infiltration, mainly lymphocytes and mononuclear macrophages, is characterized by the proliferation of small blood vessels and connective tissue, with predominant clinical changes. At the same time, the persistence of various causes leads to the coexistence of damage and repair of tissues, and in the process of repair, fibrous tissues, capillaries, epithelial cells, parenchymal cells, and others proliferate, thus causing damage to the body. Chronic inflammation is difficult to treat and is at risk of recurring [25].

In a chronic inflammatory environment, tissue damage and repair are synchronized. Inflammatory mediators are caused by endogenous chemical factors, also known as chemical mediators. Such substances accumulate over a long period and damage human DNA, destabilizing the cell’s DNA, triggering gene mutations, and leading to the development of tumors.

Therefore, to stop the development of tumors, which can cause harm to the body, it is possible to start with inflammation to treat the symptoms. Based on the current research on hydrogen and ROS, there are several strategies for treating inflammation, as follows.

#### 2.3.2. Prevention and Intervention of Hydrogen on Inflammation

According to the experimental results, hydrogen has a robust reducing ability and can selectively inhibit peroxide nitrite, hydroxyl, and other harmful free radicals. Thus, it regulates gene expression, reduces oxidative stress, and inhibits inflammatory cytokines, hormone gene expression, and other functions. Also, hydrogen is very safe. So, hydrogen provides prevention and intervention in chronic inflammatory diseases with significant advantages. It can be manifested in the following ways:

(1) In the study of animal models with yeast polysaccharide-induced systemic inflammatory response, it was found that inhalation of hydrogen can reduce organ damage in mice and improve the survival of mice. Its mechanism of action involves decreasing oxidative damage products and increasing SOD, pro-inflammatory cellular factor (HMGB1) levels [26]. Han et al. demonstrated, through cellular and animal experiments, that hydrogen energy reduces the level of phosphorylated expression of kinases (ERK) regulated by extracellular signals. This reduction, in turn, lowers the expression of inflammatory mediators, thereby attenuating the inflammatory response [26,27].
(2) In clinical observations of renal ischemia-reperfusion injury, hydrogen has been found to protect the kidneys by inhibiting many factors associated with inflammatory expression and exerting a protective effect on the kidneys. Hydrogen inhibits tumorigenesis, development, and metastasis despite the mechanism, thus inhibiting and reducing the inflammatory response [28].
(3) Inhibition of tumor cells is another advantage. Hydrogen molecules can inhibit tumors—the mechanism is often thought to be due to their antioxidant and anti-inflammatory effects. -OH and peroxide nitrite anions (ONOO-) are the body’s most toxic and potent oxidizing agents. They affect nucleic acids, lipids, and proteins, leading to DNA damage, lipid peroxidation, and protein degradation. Smoking, air pollution, chemical substances, mental stress, inflammation, and so on can induce this peroxide, prompting cells to mutate, thus producing cancer cells. Hydrogen is a specialized cleaner for two different oxidizers. This active oxygen can only be neutralized and will not affect oxygen, nitric oxide, hydrogen peroxide, or other active oxygen beneficial to the human body.

### 2.4. Application and Prospects of Hydrogen Antitumor and Current Problems

#### 2.4.1. Effects of Oxidative Stress on DNA

A healthy body produces many cancer cells daily; standard mechanisms can remove some. Tumor progression is a multistage process defined by initiation, promotion, and progression, and oxidative stress interacts with all process stages [29]. ROS can initiate tumorigenicity by inducing DNA damage and promoting subsequent tumor progression. During the final stages of cancer development, oxidative stress promotes DNA changes and development by initiating cell population. The following study addresses how to minimize the effects of ROS.

#### 2.4.2. Effects of Hydrogen-Rich Water on Oxidative Stress

(1) Hydrogen-enriched water fights ROS

Neutral pH-H_2_-enriched electrolytic water (NHE) is an antioxidant that counteracts ROS, slows tumor cell proliferation and invasion of tumor cells, and scavenges intracellular oxidants.

For example, NHE water slows the clonal growth of human tongue cancer cells and attenuates the invasion of human fibrosarcoma cells while inhibiting intracellular oxidants and scavenging intracellular oxidant H_2_ peroxides [29]. Hydrogen-rich water has been reported to remove ROS essential for cell growth, resulting in a decrease in the number of Ehrlich ascites tumor cells, apoptosis, cell shrinkage, cell deformation, and an increase in membrane microvilli.

(2) Hydrogen-enriched water reduces the biological response to oxidative stress

Oxidative stress arises in the body when excessive oxygen free radical production occurs, leading to a chain of inflammatory reactions causing cell and tissue damage [30]. Oxidative stress is widely acknowledged as the primary cause of numerous chronic ailments, including cancer. Hydrogen-rich water effectively combats oxidative stress by supplying a profusion of hydrogen molecules. Hydrogen molecules are small enough to pass through cell membranes effortlessly and enter the cell interior. Once inside, these molecules neutralize oxygen free radicals, thus reducing damage caused by oxidative stress. In addition, hydrogen-rich water stimulates antioxidant enzyme activity within cells, further improving antioxidant capacity [31]. Studies indicate that patients with malignant liver tumors who were administered hydrogen-rich water alongside undergoing radiotherapy for six weeks had substantially higher quality of life (QOL) scores and significantly lower levels of reactive oxygen metabolites in their blood [29].

#### 2.4.3. Problems with Hydrogen Antitumor

The anticancer mechanism of hydrogen can be understood from two different perspectives. Hydrogen generates an antioxidant effect, which can counteract ROS-induced side effects. Hydrogen can reduce inflammation, allowing it to more effectively combat tumor cells and potentially prevent cancer through this dual action. Research into developing and preserving hydrogen is still lacking, hampering its practical application in treatments. Despite this, hydrogen is a safer and more effective alternative to early radiotherapy with a higher risk of harmful side effects. Ultimately, using hydrogen as a novel drug can pave the way for new approaches to tumor suppression and cancer mitigation.

## 3. Carbon Dioxide (CO_2_)

### 3.1. Antitumor Mechanisms of CO_2_

#### 3.1.1. Carbon Sources for Mammals

By breaking down food into carbon dioxide, mammals gain a source of energy, and dextrose is usually thought to be metabolized through the glycolysis of tricarboxylic acid (TCA) cycle synergistically catabolized in cells. In the organism, cells and tissues can still help each other to share the metabolic tasks by exchanging intermediate products of metabolism (for example lactic acid). Therefore, Sheng Hui and others systematically investigated the significance of different circulating metabolic intermediates and found that if there is to be a profound effect on the internal fluxes of the body’s organs, it is necessary to ensure that there is a reasonable concentration of metabolites in the blood; for example, mice with 39 metabolites at sufficient concentrations (>30 μm) are likely to have more than 10% glucose.

**Figure 3:**
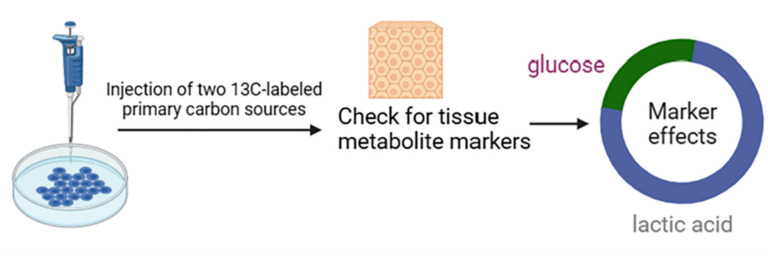
Methods for detecting significant sources of carbon.

Although glucose is widely recognized as a significant cyclic carbon source, lactate shows a 2.5-fold cyclic turnover flux on a molar basis. In order to test the hypothesis that lactate is the main circulating carbon carrier, tissue metabolite labeling was examined after a uniform injection of two main carbon-13-labeled carbon sources (glucose and glutamine) or lactate in cell culture (Figure 3). As expected, glucose infusion was best for standardizing glycolysis and pentose phosphate intermediates in tissues. In contrast, glucose and lactate tracer infusions were effective for standardized labeling of tissue lactate. In most tissues, lactate infusion works best for standardized labeling of TCA intermediates, such as malate and succinate. In the margin of error, the organism can rely on labeling circulating lactate in all tissues except the brain to account for TCA labeling in glucose. Thus, in the unfed state, most of the glucose circulation in all tissues, excluding the brain, relies on the lactate cycle to supply the TCA circuit [32].

By analyzing the experimental data, it was found that the significant source of TCA intermediates is circulating lactic acid. Another conclusion drawn from quantitative modeling is that in most tissues and genetically engineered mouse model (GEMM) tumors, the contribution of glucose to the TCA cycle is mainly via the lactic acid cycle. In the classic lactic acid cycle (Cori cycle), muscles produce lactic acid, which is then absorbed in the hepatic system for the production of glucose. Previous studies have emphasized the prospects for the development of carbon transport in inter- and intra-tissue lactate, respectively [32]. This shuttle is the basis for most circulating lactate conversion, and glucose is fed to TCA metabolism primarily through circulating lactate.

#### 3.1.2. Effect of Carbon Sources on Tumors

Lactate and glucose redox balance. To increase the NAD+/NADH ratio of all tissues in the homeostatic cell, it is necessary to accelerate the rate at which tissue lactate and pyruvate in the circulation complete their exchange, which, in turn, gives the body a buffering effect against disturbances of NAD(H) in any particular position. Because all tissues can almost exclusively commune lactate with each other, this successfully separates glycolysis and the TCA cycle within individual tissues, which, in turn, allows for uniquely independent tissue-specific regulation of these two processes. Since almost all ATP is produced in the TCA cycle, every tissue can derive power from the predominant caloric component of food (carbohydrates) without the need for glycolysis. In contrast, supporting cell proliferation and achieving NADPH production from the pentose phosphate pathway, brain activity, and whole-body glucose homeostasis can also be accomplished by regulating glycolytic activity.

Of interest, experimental data suggest that circulating lactate supports tumor TCA intermediates approximately twice as much as glucose, and quantitative analysis revealed that glucose contributes to the tumor TCA cycle only by circulating lactate. In undamaged lung tissue and lung cancer, lactate is the predominant TCA proponent. In contrast, in pancreatic cancers, the effect of glutamine is greater, consistent with the substrate preference of tumors reflecting their original tissue [32].

### 3.2. PLGA Nanoparticles for CO_2_ Generation

Because many of the chemotherapeutic anticancer drugs currently being used on a wide scale are unable to accurately distinguish between cancer cells and normal cells, the body can experience a variety of adverse reactions, such as depression, nausea, and hair fall. Based on the above problems, researchers have consistently proposed the use of polymer nanoparticles to overcome these drawbacks. The use of polymer nanoparticles allows for the introduction of targeting ligands (for example, peptides or antibodies), which, in turn, allows for the efficient and specific delivery of the contained anticancer drug to the target cell. In general, cancer sites exhibit low pH, high aerobic glycolysis, hypoxia, and angiogenesis. Cancer sites exhibit low pH because of increased lactate production due to the upregulation of glycolysis, which can be exploited to construct pH-responsive anticancer therapies [33].

Building on existing research, Hyun Jin Jang and others designed nanoparticles that may contain multiple drugs. These particles have the feature of being modifiable with various ligands against the target cancer to be treated. Hyun Jin Jang and others successfully prepared PLGA nanoparticles containing mineralized calcium carbonates (mCNPs). Adjusting the pH to acidic resulted in a faster rate of carbon dioxide gas production, which increased the rate of DOX release from the nanoparticles. In the loaded mouse model designed to enhance the aggregation of nanoparticles at sites with cancer, RVG peptide modification of mCNPs can be used. Intravenous injection of RVG-mCNP-DOX into mice with cancer effectively reduces the size of the tumor and slows down the cancer. The advantage of this method is that it does not cause weight loss in the mice, which would affect their life safety. By histological analysis, RVG-mCNP-DOX has been found to be effective in inducing apoptosis of tumor cells, which has a significant effect on cancer treatment.

### 3.3. CO_2_ Transport in Humans

Existing clinical trials have demonstrated that the easiest and simplest way to use gases for the purpose of gas therapy for cancer is to inhale them. It must be taken into account that many gases are highly irritating to the tissues where exhalation and inhalation take place. The gases also produce strong irritation in the bloodstream, as they are insoluble in the bloodstream, and these difficult-to-control drawbacks limit the systemic route of administration. While carbon dioxide does not produce any cancer treatments on its own, the production of carbon dioxide bubbles can encourage the carbon dioxide nanogenerator to release the drugs inside it, and on top of that, it can also enhance bio-imaging, which facilitates subsequent observation. Various stimulus-responsive CO_2_ nanogenerators have now been developed and they use polymers, liposomes, and other nanomaterials as nanoscaffolds [34].

#### 3.3.1. CO_2_ Transport Completed by Internal Stimuli

Suitable acidic conditions can effectively stimulate the production of carbon dioxide bubbles. Therefore, Sung and others developed a hollow polylactic acid ethylenediamine microsphere carrier loaded with DOX and sodium bicarbonate (SBC), which can generate carbon dioxide bubbles. In a separate study, Sung and others further developed a hollow PLGA microsphere carrier that could respond to ROS, which was used to complete the CO_2_ bubble-mediated drug release process.

In near-term studies, a variety of endogenous stimulation modalities have been utilized to combat cancer. However, due to the limited amount of TME endogenous stimulation, which inevitably leaks prematurely, the ability to burst carbon dioxide release within a cancer lesion remains challenging and requires further research [34].

#### 3.3.2. CO_2_ Transport Completed by Exogenous Stimulus

Stimulus-responsive CO_2_ bubble-generating liposome systems have now been successfully constructed for use in specific chemotherapy for the treatment of cancer, and the systems can be triggered by a slight elevation of temperature followed by light radiation or ultrasound. For example, Sung and others have wrapped gold nanocages containing DOX and ammonium bicarbonate (ABC) in lipid particles to locally convert near-infrared light into heat, decompose ABC to produce CO_2_ bubbles, and trigger DOX release. Polypropylene carbonate (PPC)-based block polymer micelles have been constructed that contain gold nanorods exposed to near-infrared (NIR) radiation for the generation of carbon dioxide microbubbles [34].

Relying on both internal and external stimuli can trigger and enhance the production of carbon dioxide gas or the generation of carbon dioxide bubbles, providing a new and effective way to treat cancer.

### 3.4. Study of Nano Preparations for Delivery of CO_2_

The construction of carbonate copolymer nanoparticles (Gas-NPs) generating carbon dioxide has now been realized. The successful discovery of Gas-NPs can enhance the physicochemical properties within the organism, on which nanoparticle-based anticancer drug carriers are dependent, and can improve the ability to target cancer therapy. Guided by the chemical gas generation mechanism of solid-phase carbonate copolymer precursors, simple Gas-NPs or Gas-NPs loaded with anticancer drugs were simply prepared by the oil-in-water (O/W) emulsion method, and the construction of therapeutic nanoparticles for the treatment of cancer has been successfully accomplished. The nanoscale particle structure of Gas-NPs is unaffected by the intravenous injection, remains intact, and can still be localized to patients suffering from cancer. The Gas-NPs are then hydrolyzed inside the tissue to produce a large number of voluminous carbon dioxide bubbles, which ultimately play a role in cancer therapy and contribute to the EPR effect of localizing the cancer site [35].

## 4. NO

NO is an endogenous free radical, a volatile diatomic gas molecule that plays an essential role in various physiopathological processes. It plays a vital role as a messenger in various biological processes and can induce certain physiological or biochemical changes in cells, tissues, or organisms. Due to the unique physiological regulation of NO, it may show some specific therapeutic effects on some specific diseases, such as infection, cancer, neurotransmission, etc. It has attracted widespread interest from researchers and can be protective or destructive, depending on its concentration, location, cellular environment, etc.

In terms of clinical applications, NO is recognized as a gas that modulates many cancer-related events and exhibits a dual role in cancer [36]. At lower concentrations, NO can promote tumor growth [37]. However, at higher concentrations, NO exhibits pro-tumorigenic effects through various mechanisms. At the present stage, NO, as a novel therapeutic agent, has been widely used for exogenous and in vivo administration in cancer cells. However, NO is a free radical gas molecule with a short half-life (about six years) and a diffusion radius (40~200 μm). Wang et al. encapsulated NO into microbubbles to improve NO’s stability [38]. It is currently used in the treatment of deep vein thrombosis with excellent results. NO microbubbles do more than that—they also reduce the size of thrombi and inhibit platelet and inflammatory cell aggregation. It enters the body mainly through the NO donor [39]. NO is then released from the NO donor by different stimuli. However, there are also problems: most NO donors are unstable in the physiological environment and have a short half-life. Storing this dangerous NO donor is a complex problem. More importantly, the selective accumulation of NO in tumor tissues is extremely important when applying NO donors for cancer therapy due to the concentration-dependent dual action of NO.

The tumor inhibitory effect of NO has a strong concentration dependence. For better results, NO gas treatment is often combined with other treatments, including chemotherapy, radiotherapy, photodynamic therapy, and more.

### 4.1. Combination of NO and Chemotherapy

**Figure 4:**
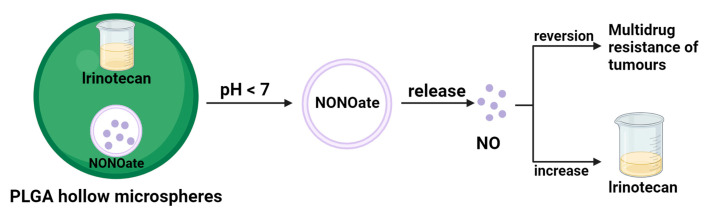
NO reverse multidrug resistance schematic.

Tumor multidrug resistance (MDR) is a major cause of failure in cancer patients undergoing chemotherapy. Given the many functions of NO in cancer therapy, researchers are increasingly interested in the potential application of NO in reversing MDR [40]. For example, Davis et al. conducted experimental studies to demonstrate that the introduction of NO during chemotherapy can enhance the anticancer activity of chemotherapeutic agents [41]. MDR is usually associated with the overexpression of integral membrane transport proteins, such as P-glycoprotein (Pgp), MDR-related proteins (MRPs), and other essential membrane transporter proteins overexpressed, leading to intracellular drug efflux to the outside. In a study, Ghigo et al. found that the addition of NO effectively inhibited the expression of Pgp and MRPs, resulting in a significant increase in adriamycin (DOX) accumulation and ultimately inhibited cell proliferation [42]. It can be concluded that reversing drug resistance in DOX cancer therapy can be achieved by NO-based gas therapy. In another study, Sung et al. prepared an injectable PLGA hollow microsphere (HM) that encapsulates the NO donor NONOate and the anticancer drug irinotecan well [43]. In an acidic environment, the PH-sensitive NONOate enhances the intracellular accumulation of irinotecan by releasing large amounts of NO, thereby reversing the Pgp-mediated MDR (Figure 4). In addition to this, high levels of intracellular glutathione are another reason for multidrug resistance. For example, the insensitivity of platinum drugs to cancer cells with high GSH levels is due to the formation of PtGSH adducts. This is where the synergistic effect of NO comes to the fore—as a free radical gas molecule, NO eliminates much of the intracellular GSH and overcomes the MDR [44]. In a representative study, mesoporous silica nanoparticles (MSN) can be loaded with NO donor and cisplatin [45]. The combination of NO gas and cisplatin is much more effective and synergistic than NO gas therapy and cisplatin chemotherapy. Therefore, NO can enhance the cytotoxicity of cisplatin and reverse multidrug resistance.

Therefore, the design and construction of a nanocarrier that can effectively encapsulate NO, and chemotherapeutic drugs can not only exert the antitumor effect of NO but also achieve the sensitizing effect of chemotherapeutic drugs at tumor sites.

### 4.2. NO in Radiotherapy

Since the 1950s, researchers have found that NO is a very effective sensitizer for radiation therapy, can act as a radiosensitizer for hypoxic cells, and is particularly effective in treating cancer [46]. Tumor hypoxia and high intracellular glutathione levels are two significant obstacles to tumor radiotherapy. However, it is worth noting that NO depletes NO in human cells and improves oxygenation through vasotonic effects, and many NO donors are sensitive to X-rays. Therefore, X-ray radiation can sensitize radiotherapy by efficiently releasing NO. Typical examples that have appeared in the study are silica shell-coated UCNPs (USMSs), prepared by covalent coupling of the NO donor SNO and biocompatible PEG-constituted PEG-USMSsSNO [47]. After X-ray irradiation, due to the high tissue penetration of X-rays, NO is released from PEG-USMSs-SNO in a nondependent manner. Most importantly, the release of NO, intracellularly and in vivo, can be controlled by the degree of X-ray radiation exposure. In addition, the released NO has a strong radiosensitizing effect, with the result of killing cancerous cells and inhibiting their growth. Policastro L et al. evaluated the radiosensitization of NO concerning the degree of malignancy [48]. After irradiating cancer cells of different degrees of malignancy with diethylenetriamine (DETA)-based NO donor, DETA-NO, and gamma rays, the surprising funding was that DETA-NO exhibited better radiosensitization of the more cancer cells, which is of great significance for the practical application of NO in radiotherapy.

### 4.3. Combined NO and Photodynamic Therapy

Photodynamic therapy is a minimally invasive therapy that produces cytotoxic ROS. Photodynamic therapy must be performed under the simultaneous presence of three conditions: photoexcitation, oxygen molecules, and photosensitizers in the range of visible light to near-infrared light. To generate cytotoxic ROS, photodynamic therapy must have the simultaneous presence of three requirements: light excitation in the visual to the near-infrared range, oxygen molecules, and photosensitizers before the therapy can destroy proteins, unsaturated lipids, and nucleic acids in the target tumor cells. In their research, Ahmad et al. found that NO could participate in photodynamic therapy-mediated cell cycle block and cause apoptosis [49]. In addition, NO increased the sensitivity of cancer cells to ROS and reacted with ROS to produce toxic substances such as N_2_O_3_ and ONOO-. Under active conditions, ONOO- can also generate OH and NO_2_ radicals by cleavage and interacting with CO_2_ to produce another strong oxidant, CO^3−^, which provides a theoretical basis for NO gas-sensitizing photodynamic therapy. Therefore, combining photosensitizers with NO donors is expected to be a more desirable tumor therapeutic tool.

Although there are still some unresolved issues in the process of NO anticancer activity, NO gas therapy is worth exploring further as a potentially valuable therapeutic approach. 

## 5. Carbon Monoxide Anticancer Therapy

Carbon monoxide (CO), a gaseous molecular medium, is recognized as a toxic gas because of its higher affinity for hemoglobin than for oxygen. Meanwhile, biological studies have revealed that CO is an endogenous signaling molecule continuously produced in the healthy human body, which possesses a variety of cellular activities, including anti-inflammatory, antiapoptotic, and antiproliferative activities [50]. The toxicity of CO and the way it is administered should be strictly controlled; otherwise, it may easily threaten the health of patients and healthcare workers. In addition to safety issues, there are other difficulties and problems with this type of drug delivery. The main one is the need for more tissue-specific delivery. After CO enters the body and circulates through the bloodstream, it is impossible to distinguish between healthy and pathological parts of the patient, which is extremely dangerous. Due to the relatively low water solubility of CO, it cannot be targeted to specific cells or tissues when administered, and the amount released cannot be effectively controlled [51]. In recent years, a series of compounds have been fabricated to release CO into biological systems in a controlled manner in space and time known as CO-releasing molecules (CO-RMS). Therefore, CO-RMS is considered a convenient and safe mode of CO delivery and has emerged as the best alternative to gas drug delivery in clinical practice [52].

### 5.1. Anticancer Activity of CO-RMs Targeting Different Cancers

In layman’s terms, CO-RMs refer to those substances that are managed and dispersed in the context of biological systems. In the system, they are induced to operate and then activated biologically by releasing CO in a stable and controlled manner into diseased target tissues and organs. Structurally, CO-RMs typically comprise two important parts surrounding the core: the CORM sphere and the drug sphere (for example, the metallic base of the CO-RM contains transition metal centers). CO-RM spheres, or coordination spheres, comprise several CO ligands and an internal part defined in a spatial arrangement. In terms of action, it has a decisive effect on the chemometrics, associated dynamics, and initiating machinery of CO release. Meanwhile, the pharmacological properties of CO-RM action are determined by the fact that the drug spheres are located in an external position, defined by the periphery of the co-ligand, which induces tissue-specific targeting by modulating the distribution ratio between body fluids and tissues [53]. Theoretically, by appropriately adjusting the ratio of CORM spheres to drug spheres to the desired range, the engineered CO-RM can be used for a variety of specific biomedical applications.

Because of the long survival cycle characteristic of cancer cells, an effective way to treat cancer is to induce targeted apoptosis, which results in the programmed death of cancer cells [54]. In current studies targeting cancer, CORM-2, a tricarbonyl ruthenium (II) chloride dimer, is the most widely used substance for clinical applications of CO-RMs. In relevant literature reports, CORM-2 has been shown to have antiproliferative and proapoptotic effects on various cancer cell types, such as breast, prostate, colon, and colorectal cancers, lung, stomach, pancreatic and lymphoma cancers, etc. Meanwhile, the significant inhibitory effect of CORM-2 on tumor cell growth has also been amply demonstrated in in vitro clinical trials in different hormonal rodent models [55]. In the opposite direction, human hepatocellular carcinoma cell systems were placed in an in vitro setting and the results showed that CORM-2 pretreatment interrupted the inhibitory effect on cancer cell growth by attenuating cell cycle arrest [56]. These conflicting studies amply demonstrate that CO-RMs contribute to determining the destiny of cancer cells by specifically influencing cell proliferation and death.

Another distinguishing feature of cancer is metastasis, the transfer of cancer cells from their original site to neighboring sites and remote organs. An important cause of cancer development and death is reported to be metastasis, which accounts for 90% of total cancer fatalities [57]. Consequently, cancer treatment can be achieved by preventing cancer metastasis. Of interest, it was experimentally demonstrated that CORM-2 had a significant inhibitory effect on the migration and infiltration of colon cancer Calu-3 cells. Similarly, a recent study reported that CORM-2 encapsulated by a styrene-maleic acid copolymer inhibited the migration and invasion of two types of colorectal cancer cells [58]. Taken together, these preliminary studies suggest new potential applications of CO-RMs concerning cancer, i.e., protecting the body from metastasis by inhibiting the ability of cancer cells to invade surrounding sites.

In summary, CO-RM releases CO in the presence of CO for the treatment of a variety of cancer models, including those of the breast, prostate, colon, cervix, stomach, pancreas, skin, and lymphoma. In response to some important characteristics of cancer, although CO-RMs differ in their anticancer active effects and have cell group specificity, they can still be one of the potential anticancer drug candidates, and more studies are needed to prove this.

### 5.2. Mechanisms behind the Anticancer Activity of CO-RMs

In terms of anticancer mechanisms, CO-RMs can fight cancer by regulating ROS. First, CO-RMs can downregulate ROS to suppress tumors. Mitochondria, as a significant source of intracellular ROS production, are also an important target for the drug delivery effects of CO. However, administration of CO impairs mitochondrial function and respiration, thereby inducing downregulation of increased ROS production. One of the critical pathways abnormally activated in many cancers is the phosphatidylinositol 3-kina-se/protein kinase-b/mammalian target of rapamycin (PI3K/Akt/mTOR) signaling cascade, which results in varying degrees of interference with cell proliferation and regulation of various cellular processes [59]. Preliminary studies have shown that CO released via CORM-2 disrupts the PI3K/Akt/mTOR pathway to achieve the goal of inhibiting abnormal cell proliferation and overgrowth in the same allogeneic lung tumor mouse model. A decrease in the phosphorylation of PI3K, Akt, and mTOR phosphorylation clinically characterizes it. The downstream effector of mTOR phosphorylation, p70 S6 kinase, which promotes sustained cellular growth and proliferation, is also downregulated by CORM-2. Meanwhile, further colorectal or pancreatic cancer studies found a significant decrease in Akt phosphorylation after CORM-2 treatment [60]. In summary, CO-RMs inhibit the PI3K/Akt/mTOR pathway, thereby suppressing cancer tumor cell multiplication signaling. Second, CO-RMs can also induce apoptosis by the upregulation of ROS. In particular, high concentrations of CO can inhibit mitochondrial respiration and modulate ROS production to cause oxidative stress, activating the apoptotic pathway [55].

**Figure 5:**
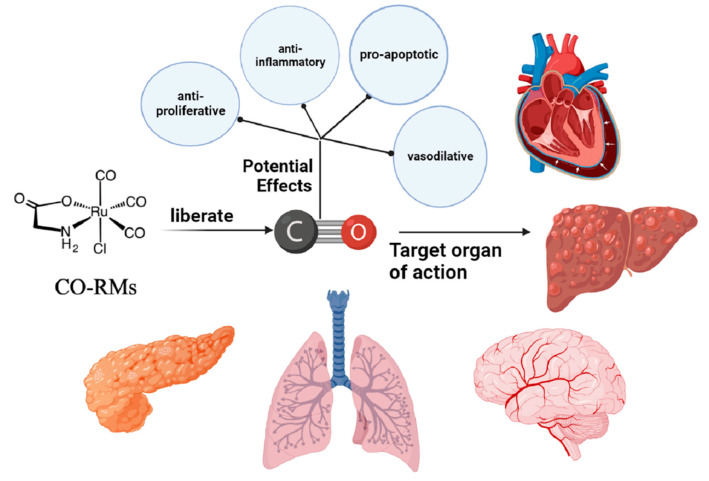
Mechanism of anticancer activity of CO-RMs.

The antitumor mechanism of CO has yet to be fully defined. Mechanistically, ROS may be centrally located, and a complex signaling pathway supports the anticancer activity of CO-RMs. It is important to note that ROS faces great challenges in cancer therapy due to its dual role—upregulating or downregulating ROS production can affect cancer progression. The current study has no direct evidence for a direct relationship between ROS signaling molecules and CO release from CO-RMs, which is a significant limitation. Finally, the delivery of CO via CO-RMs, despite its proven benefits in various cancer models in vivo and in vitro, has proven benefits across multiple cancer models in vivo. However, there are still some barriers to the feasibility and applicability of CO-RMs in clinical aspects of therapeutic medicine. Developing CO-RMs that meet the safety and suitability requirements for clinical use is a crucial direction for future research (Figure 5).

## 6. Hydrogen Sulfide

H_2_S is a colorless, toxic, corrosive gas that gives rotten eggs their characteristic odor. For a long time, H_2_S has been recognized as an environmental toxin and biohazard with a wide range of toxicological effects on mammals, including humans. However, it was recognized as a third gas transmitter in 2002 [61]. Similar to the other two gaseous transmitters, nitric oxide (NO) or carbon monoxide (CO), H_2_S plays a vital role as a mediator in a wide range of physiological processes and has a crucial role in the establishment and development of a wide range of health disorders, from noncancerous to cancerous [62,63].

### 6.1. Progress in Research

#### 6.1.1. Mechanism of H_2_S

H_2_S plays an important role in many physiological processes, such as muscle relaxation, water regulation, cytoprotection, and inflammation [64,65,66,67]. A study of patients with hepatocellular carcinoma (HCC), analyzing their tissue samples, found that cystathionineβ-synthase (CBS) levels were significantly lower in hepatocellular carcinoma samples relative to surrounding noncancerous cells, and that this reduction may be related to recovery from surgery [68]. Further downregulation of CBS enzymes using inhibitors induces anticancer effects through iron apoptosis, which is an iron-dependent cause of programmed cell death in HepG2 cells [69]. In contrast to CBS, cellular cystathionine gamma cleavage enzyme (CTH) expression is upregulated in a wide variety of cancer types, including HCC, while endogenous inhibition, which exerts one of its anticancer effects, has also been demonstrated [70]. At the same time, it has been demonstrated that donor-supplemented H_2_S can also have a protective effect on effective cells in many cancer types [71]. Despite the incomplete understanding of the normal range of H_2_S in various types of cells and the alterations that make it active in cancer cells, it is clear from the available databases that only a certain amount of H_2_S needs to be maintained in order to maintain cellular activity. Still, any poorly mastered adjustment can dramatically impact the cellular activities involved in cancer regulation [72].

#### 6.1.2. Role of H_2_S in Cancer

(1) Tumor growth in progress

Elevated enzymes of H_2_S have been observed in many types of cancers, and tumor growth in cancers such as colon and lung cancer has been inhibited due to a decrease in CBS or CTH activity [73,74]. MEK1 belongs to the classical MAPK kinase pathway, and its activation is synonymous with cell proliferation and tumor growth [75]. Therefore, a key driver of tumor growth in CBS or CTH overexpressing tumors may be the ability of H_2_S-mediated MEK1 persulfuration to stimulate ERK1/2 activity [76].

(2) In-cancer metabolism

Exogenous H_2_S affects ATP production by inhibiting mitochondrial complex IV and, thus, mitochondrial electron transport; however, endogenous H_2_S does not act the same way as exogenous H_2_S [77]. The primary function of mitochondria is to provide ATP, and endogenous H_2_S is taken up and oxidized by the mitochondria, thus facilitating the synthesis of aerobic respiratory ATP [77]. Although it is uncertain whether this phenomenon contributes to cancer remission, it is possible to make a slight guess that tumor cells can only produce ATP via this pathway when well oxygenated [78]. Tumor cells inevitably develop without the necessary nutrients to maintain their viability and proliferation and to enhance their proliferation ability; tumor cells will preferentially convert glucose to lactate through aerobic glycolysis regardless of oxygen availability [79]. Cancer cells use lactate dehydrogenase A (LDHA) to increase the rate of glycolysis to carry out an endless program of cell proliferation. At the same time, the enzymatic activity of LDHA has been identified as a therapeutic target to inhibit the growth of different types of tumors and distant metastasis [80]. H_2_S-mediated per sulfuration of LDHA at Cys 163 enhances its enzymatic activity, leading to an increase in lactate production in HCT116 colon cancer cells, which is accompanied by a decrease in oxygen depletion and ATP production, representing the possibility that H_2_S can regulate cancer metabolism to cause uncontrolled tumor cell growth.

### 6.2. H_2_S Gas Therapy

In recent years, the impact of H_2_S donors in many cancer types has been more or less recognized, contributing to cancer progression [72]. It is understood that higher levels of H_2_S produced by donors can be used as an anticancer drug to induce uncontrolled cell acidification, resulting in apoptosis and cell cycle arrest [71,81]. Although scholars still have some controversy about its use in the study of cancer, it can be explicitly explained by the bell-shaped model, which was used by Hellmich and Szabo in their modeling experiments to show that low concentrations of H_2_S have pro-cancer effects, while in contrast, high concentrations of H_2_S have anticancer properties [82,83].

**Figure 6:**
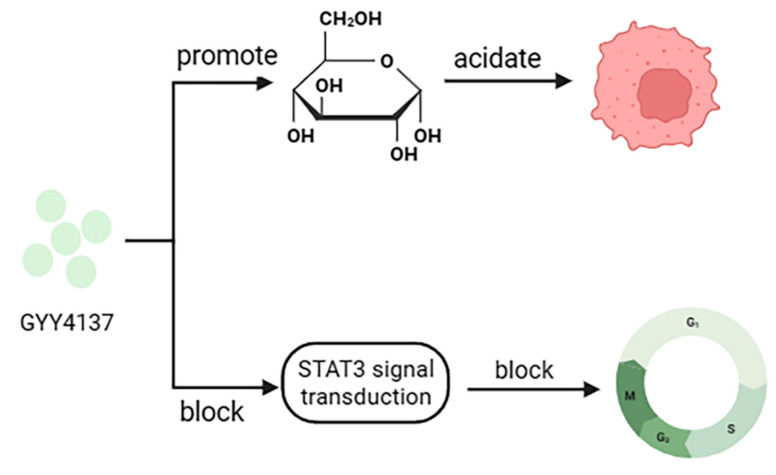
Anticancer mechanism of GYY4137.

A variety of H_2_S donors have now been prepared and can achieve cancer cell death when clinically tested, provided there are high concentrations or prolonged exposure conditions [84,85]. GYY4137 was created as a slow-release H2S donor that promotes glucose uptake, glycolysis, and lactate production and decreases the activity of pH regulators, anion exchangers (AE), and sodium/proton exchangers (NHE), leading to intracellular acidification in cancer cells [81]. In addition, GYY4137 can block STAT3 signaling, leading to cell cycle arrest, apoptosis, and inhibition of hepatocellular carcinoma tumor growth (Figure 6) [84]. However, the drawback is that the toxicity of these H_2_S donors in normal cells is unavoidable, which is the main problem facing the current development of H_2_S donor drugs.

In summary, with its diverse mechanisms, gas therapy will provide a greater variety of agents and therapeutic modalities (Table 1) for cancer patients and is of high research value.

## 7. Sulfur Dioxide (SO_2_)

Sulfur dioxide has traditionally been regarded as an air pollutant. Studies indicate that inhaling SO_2_ at elevated concentrations has a potent toxicity that can lead to oxidative stress-mediated harm to biomolecules such as proteins, lipids, and DNA [90,91]. Recent reports suggest that SO_2_ plays a significant role in fighting diseases, notably its potential as a vasorelaxant and antibacterial agent [92,93]. Meanwhile, in collaboration with others, SO_2_ may serve as an auxiliary gas to overcome cancer chemotherapy resistance [94]. Nevertheless, its poor biocompatibility and limited accumulation and release during treatment in organisms have become limitations to its application for cancer treatment in vivo [95,96]. In the context of this study, the establishment of a therapeutic system that allows for efficient release and restrained generation of SO_2_ could offer a promising platform for treating cancer. However, the potential side effects need to be explored. Consequently, there is an opportunity to further examine the ex vivo and ex vivo therapeutic impacts of SO_2_ on tumor treatment.

### 7.1. Combination of Sulfur Dioxide and Chemotherapy

Melanoma, a malignant tumor resulting from abnormal melanocyte proliferation, is a frequently encountered clinical tumor with a rapidly increasing incidence rate. There is an urgent need for effective treatment to reduce the incidence of melanoma. Currently, melanoma treatment still relies on chemotherapy. In recent years, SO_2_ has garnered significant attention for its ability to inhibit tumor growth. An et al. developed a new SO_2_ polymer precursor capable of loading adriamycin (DOX) to form drug-carrying nanoparticles (PDDN-DOX) [87]. This innovative solution shows promise for improving melanoma treatment. After internalization by tumor cells, PDDN-DOX released both SO_2_ and DOX in the presence of glutathione, thereby suppressing melanoma cell proliferation and significantly inhibiting tumor growth in subcutaneous and metastatic melanoma mouse models. Therefore, SO_2_ gas therapy combined with chemotherapy represents an efficacious therapeutic strategy for treating subcutaneous and metastatic melanoma.

### 7.2. Design of Sulfur Dioxide Prodrug

Sulfur dioxide, a gas with strong irritant properties for the respiratory tract, can be challenging to manage. As a result, various inorganic sulfites and organic SO_2_ donors are frequently employed as substitutes. Ion pairs are the most widely used source of SO_2_ in therapeutic applications [34]. Complex transformations occur between hydrated SO_2_•H_2_O and ions, resulting in difficulties when calculating the release rate of SO_2_. To tackle the issue of uncontrollable SO_2_ release, Chen and colleagues have developed a SO_2_ precursor molecule that enables precise control over the rate and quantity of SO_2_ released. This has been achieved by merging a dienophile group (alkyne) and a dienophile group (thiophene s-2) in a single molecule, leading to the spontaneous release of SO_2_ in physiological conditions through an intramolecular counter-electron-demand Diels–Alder reaction [34]. The authors have also investigated the effects of different reaction conditions on the SO_2_ release rate and yield [34]. Ji and colleagues used intramolecular cycloaddition reactions to develop a range of SO_2_ precursors with adjustable release rates and half-lives from minutes to days [88]. Wang and team also synthesized a series of esterase-sensitive SO_2_ precursors by constructing an intramolecular cycloaddition reaction of thiophene SO_2_ with strain alkyne to improve the control of SO_2_ release rate [89].

## 8. Challenges and Perspectives

The inherent properties of gas molecules are intricate, and the mechanisms that govern them within organisms are varied. Nonetheless, the clinical application of gas therapy is significantly impeded by the low solubility and high diffusivity of the molecules and their inadequate tissue penetration. To overcome this hurdle, integrating gas therapy with other therapies presents a promising approach to cancer treatment. Light therapy combined with gas therapy is currently a popular direction for research. Light therapy, comprising mainly photodynamic therapy (PDT) and photothermal therapy (PTT), is a therapeutic approach that invades cancer cells. This generates cytotoxic ROS under light or thermal conditions, causing irreversible cellular damage and inducing cell death. The combination of gas therapy and phototherapy aims to achieve a therapeutic effect that exceeds their individual effects. An in-depth investigation of the inherent relationship between phototherapy and gas therapy is essential to fully exploit the advantages of these therapeutic modalities. The rapid advancement of synergistic therapy has led to the emergence of numerous multifunctional nanodiagnostic systems that employ nanomaterials to enhance gas molecule delivery and promote gas molecule release through endogenous or exogenous stimulation at the point of disease to achieve therapeutic efficacy. However, it is essential to focus on developing nanosystems with simpler structures and fewer components to reduce uncertainty in vivo, given the complexity of biological systems and the metabolism of nanomaterials. In brief, the local administration of NO, CO, H_2_S, and SO_2_ in high concentrations to tumors might have inhibitory or cytotoxic effects. Still, the inherent toxicity of these molecules is significantly lower than that of most “professional” chemotherapeutic agents. Based on the varied physiology of gas signal molecules, the amalgamation of gas therapy with chemotherapy, immunotherapy, radiotherapy, targeted therapy, or phototherapy is anticipated to foster the clinical utilization of gas therapy and is crucial in enhancing the efficacy of cancer treatment.

## 9. Conclusions

Gas therapy is an emerging and promising approach to treating cancer. This review summarizes the research progress in anticancer gas therapy during the last decade, covering H_2_, CO_2_, NO, CO, H_2_S, and SO_2_ gases. However, the clinical use of gaseous nanomedicines remains very limited. On the one hand, the carcinogenicity of gases varies, and the biological mechanisms of different gas molecules against cancer need further exploration for establishing dose-response relationships. The therapeutic effect of single gas therapy is restricted. Therefore, it is recommended that diversified gas combination therapy strategies and drug delivery modes are constructed reasonably to optimize therapeutic effects and decrease side effects on normal cells and tissues. This will further enhance clinical application. To conclude, gas therapy shows promise as a cancer treatment strategy that warrants exploration and development.

## Figures and Tables

**Table 1:** The current research progress and applications of the leading gases in treating malignant tumors.

Gas Classification	Combined Therapeutic Approach	Treatment Strategies	Therapeutic Effect	Ref.
NO	Chemotherapy	Construction of a nanocarrier capable of efficiently encapsulating NO and chemotherapeutic drugs	Exerts the antitumor effect of NO itself, achieving sensitization of chemotherapeutic agents at the tumor site.	[41,42,43]
	Radiotherapy	NO release can be controlled by the level of X-ray irradiation	The released NO has a strong radiosensitizing effect, thus killing cancer cells and inhibiting tumor growth.	[48]
	Photodynamic therapy	NO enhances the sensitivity of tumor cells to ROS and reacts with ROS to generate highly toxic substances such as N_2_O_3_ and ONOO-	Photodynamic therapy destroys proteins, unsaturated lipids, and nucleic acids in tumor cells at the target site. A combination of photosensitizer and NO donor has higher antitumor ability.	[49]
CO	CO-RMs treatment method	CO-RMs release stable and controllable CO gas in vivo to diseased target tissues and organs as a way to stimulate biological anticancer activity	There are corresponding mechanisms for different cancer cells, and they can also regulate ROS to fight cancer by disrupting abnormal cancer activation pathways or inducing apoptosis in cancer cells.	[52,55]
H_2_S	H_2_S donor therapy	Construction of donors that can release high concentrations of H_2_S in vivo	Higher levels of H_2_S produced by donors act as anticancer agents to induce acidification of uncontrolled cells, resulting in apoptosis and cell cycle arrest.	[71,81,84,85]
H_2_	ROS anticancer therapy	Antitumor by lowering or elevating ROS	Antioxidants inhibit ROS-induced oxidative DNA damage and suppress tumor cell growth. By studying the ROS metabolic pathway, it is possible to reduce its adverse effects and inhibit tumor resistance.	[21]
	ROS response to inflammation	ROS inhibit inflammatory response and attenuate immune system attack on pathogens	ROS removes dead cells from the body and prevents inflammation from occurring. This prevents inflammation from developing into tumors. The prevention of tumor occurrence is the root of the problem.	[86]
	Effects on inflammation	H_2_ has strong reducing properties and selectively inhibits some harmful free radicals	The reducing nature of H_2_ gives it the ability to regulate gene expression, reduce oxidative stress, inhibit inflammatory cytokines, and hormone gene expression.	[26,27,28]
	Hydrogen-rich water fights ROS	Anti-ROS, slowing down tumor cell proliferation and invasion	Enriched electrolytic water (NHE) slows the proliferation of tumor cells while inhibiting intracellular oxidants.	[29]
CO_2_	PLG nanoparticles modified with RVG peptides	Enhanced accumulation of nanoparticles at tumor sites in a hormonal mouse model	Reduces tumor size and effectively induces apoptosis in tumor cells.	[33]
	Endogenous stimulus-responsive CO_2_ transport in the body	Construction of hollow PLGA microsphere carriers	Generation of CO_2_ bubbles	[34]
	Exogenous stimulus-responsive CO_2_ transport in the body	Encapsulation of gold nanocages with DOX and ammonium bicarbonate in lipid particles	Decomposition of ammonium bicarbonate at mildly elevated temperatures produces CO_2_ bubbles and triggers DOX release.	[34]
	Exogenous stimulus-responsive CO_2_ transport in the body	Construction of polypropylene carbonate (PPC)-based block polymer micelles containing gold nanorods	Generation of CO_2_ microbubbles under near-infrared radiation	[34]
	Utilizing nanomedicines with higher tumor-targeting capabilities	Construction of carbonate copolymer nanoparticles (Gas-NPs)	Gas-NPs in targeted tumor tissues can be hydrolyzed to produce a large number of microCO_2_ bubbles.	[35]
SO_2_	Chemotherapy	A new nanoparticle was created by utilizing an SO_2_ polymer prodrug with doxorubicin	Release of SO_2_ and DOX, combined with efficient inhibition of melanoma cell proliferation	[87]
	Design SO_2_ prodrug	Building SO_2_ donors for continuous endogenous sulfur dioxide production	Cytotoxic SO_2_ induces cell apoptosis.	[88,89]

## Data Availability

Data sharing is not applicable.
